# Maternal childbirth experience and time in labor: a population-based cohort study

**DOI:** 10.1038/s41598-022-14711-y

**Published:** 2022-07-13

**Authors:** Sara Carlhäll, Marie Nelson, Maria Svenvik, Daniel Axelsson, Marie Blomberg

**Affiliations:** 1 Department of Obstetrics and Gynecology, Linköping University, Linköping, Sweden; 2 Department of Biomedical and Clinical Sciences, Linköping University, Linköping, Sweden; 3 Department of Obstetrics and Gynecology, Region Kalmar County, Kalmar, Sweden; 4 Department of Obstetrics and Gynecology, Ryhov County Hospital, Jönköping, Sweden

**Keywords:** Preventive medicine, Public health, Epidemiology, Population screening, Epidemiology, Outcomes research, Reproductive signs and symptoms, Medical research, Risk factors

## Abstract

A negative childbirth experience may have long term negative effects on maternal health. New international guidelines allow a slower progress of labor in the early active phase. However, a longer time in labor may influence the childbirth experience. In this population-based cohort study including 26,429 women, who gave birth from January 2016 to March 2020, the association between duration of different phases of active labor and childbirth experience was studied. The women assessed their childbirth experience by visual analogue scale (VAS) score. Data was obtained from electronic medical records. The prevalence of negative childbirth experience (VAS 1–3) was 4.9%. A significant association between longer duration of all labor phases and a negative childbirth experience was found for primi- and multipara. The adjusted odds ratio (aOR (95%CI)) of negative childbirth experience and longer time in active labor (above the 90th percentile) in primipara was 2.39 (1.98–2.90) and in multipara 2.23 (1.78–2.79). In primi-and multipara with duration of labor ≥ 12 h or ≥ 6 h the aOR (95%CI) of negative childbirth experience were 2.22 (1.91–2.58) and 1.91 (1.59–2.26) respectively. It is of great importance to identify and optimize the clinical care of women with longer time in labor to reduce the risk of negative childbirth experience and associated adverse long-term effects.

## Introduction

The woman’s experience of care during childbirth is as important as an optimal clinical care to achieve desired outcomes of childbirth and labor according to the World Health Organization (WHO)^1^. Despite this, a negative childbirth experience has been reported in 5–10% of laboring women^2–5^ which may have long-term effects on maternal health^6^. Posttraumatic stress disorder, postpartum depression and dysfunctional bonding with the newborn have been related to a traumatic childbirth experience^7,8^. Furthermore, a negative childbirth experience may lead to secondary fear of childbirth, longer interval to subsequent deliveries and increased risk of future cesarean section (CS)^9–12^.

Maternal childbirth experience is affected by several factors including maternal age, mode of delivery, postpartum hemorrhage, low Apgar score, induction of labor and obstetric anal sphincter injury^2,3,6,13^. Emergency CS has been described as a significant risk factor for a negative childbirth experience^2,4,6,14,15^. The CS rate increases worldwide^16^. To reduce the incidence of emergency CS due to failure to progress, new guidelines on normal labor progression have been published by the WHO^1^ and the American College of Obstetricians and Gynecologists (ACOG)^17^, allowing a slower progress in the beginning of the active phase of labor compared with the traditional guidelines by Friedman^18^. However, a long time in labor may increase the risk of a negative childbirth experience, which in turn may lead to a future demand of CS in the following pregnancy^19,20^. When a study compared childbirth experience in women randomized to follow either the traditional partograph or new guidelines allowing longer time in labor, women following new guidelines scored lower on positive memories and feeling of control^21^.

The knowledge about maternal childbirth experience in relation to time in active labor is sparce and studies show conflicting results. A few studies have analyzed duration of the active labor in primiparous women, most without taking possible confounders into account, and concluded that prolonged labor was a risk factor for a negative childbirth experience^14,19,22^. Another study could not confirm that duration of the first stage of labor affected the childbirth experience but found that a longer pushing phase contributed to a negative childbirth experience^23^. Further, in a Swedish single center study the maternal childbirth satisfaction was not affected by the duration of neither the latent nor the active phase of labor^24^.

There are not only conflicting results concerning the impact of time in labor on maternal childbirth experience in the previous literature, but there is also inconsistent data on relevant adjustments related to time in labor as the exposure. Most of these studies are based on single center cohorts, increasing the risk of selection bias.

Thus, this study aimed to evaluate whether the duration of active labor was associated with the women’s self-reported childbirth experience. We hypothesized that longer time in active labor increased the risk of a negative childbirth experience.

## Methods

### Study-design and population

This retrospective population-based cohort study was conducted between January 2016 and March 2020 at all the seven delivery units in the southeast health care region of Sweden. All women giving birth to a singleton infant at or above 37 gestational weeks were included. The women with elective CS, non-cephalic presentation and stillbirth were excluded as well as the women with missing data on childbirth experience and missing labor time estimates before the final analyses.

After delivery, the women were asked by the midwife at the postnatal ward to assess their overall experience of the active phase of childbirth by using a visual analog scale (VAS) ranging from 1 to 10, where 1 is a very negative experience and 10 is a very positive experience. This assessment of satisfaction of childbirth by VAS is a well-established routine in the postnatal care at all participating delivery units included in this study. The VAS scoring is usually completed within 2 days after delivery, before discharge from the postnatal ward and is documented in the women’s electronic medical record. The overall assessment of satisfaction of childbirth by VAS has been validated and is comparable with the Wijma Delivery Experience Questionnaire (W-DEQ) and the Childbirth Experience Questionnaire (CEQ)^24–26^.

### Data collection and definitions

Data in this study was obtained from the women’s electronic medical records. The maternal variables that were extracted included the self-reported childbirth experience VAS score, maternal age at time of delivery, early pregnancy body mass index (BMI), parity and gestational age at delivery. Labor and neonatal variables assessed were onset of labor (spontaneous or induction), usage of epidural anesthesia, oxytocin augmentation, mode of birth (non-instrumental vaginal delivery, instrumental vaginal delivery, or emergency CS), obstetric anal sphincter injury, postpartum hemorrhage, Apgar score at five minutes and birth weight. Further, the time estimates for start of active labor, start of pushing contractions, and time of birth were extracted from the electronic medical records.

Maternal BMI was categorized according to the World Health Organization (WHO) classification: < 18.5 kg/m^2^ (underweight), 18.5–24.9 kg/m^2^ (normal weight), 25.0–29.9 kg/m^2^ (overweight), 30–34.9 kg/m^2^ (class I obesity), 35.0–39.9 kg/m^2^ (class II obesity) and ≥ 40 kg/m^2^ (class III obesity).

Primiparous women and multiparous women were analyzed separately. Women with induced labor and a spontaneous onset of labor were included in the analyses as well as women with emergency CS.

The main exposure was duration of labor. The different labor time variables that were analyzed were the duration of the total active labor (from start of the active labor until the time of birth), the active phase in the first stage of labor (from start of the active labor until start of the pushing efforts) and the pushing phase. Start of active labor was defined according to the Swedish nationally recommended definition, which states that at least two out of three of the following criteria must be fulfilled: spontaneous rupture of the membranes, regular painful contractions (2–3/10 min), and the cervix dilated four centimetres or effaced and dilated more than one centimetre. In addition to these criteria, the labor should progress within the following 2 h^27^. The midwife at the delivery ward prospectively documented the time when the active phase and the pushing efforts started and the time of birth. The distribution of total labor duration is presented in a descriptive approach as crude observed data of the 10th, 25th, 50th, 75th and 90th percentiles of time in active labor. For the purpose of this study, prolonged labor was defined based on the Swedish definition of dystocia (cervical dilatation less than 1 cm/hour during the active phase and > 3 h from cervix fully dilated to delivery for primiparous women)^28^ and previous similar research, defining prolonged labor ≥ 12 h for primiparous women^14,19^ and clinically relevant cut offs. The total active labor was defined prolonged if duration was ≥ 12 h in primiparous women and ≥ 6 h in multiparous women. The active phase of the first stage of labor was defined prolonged if duration was ≥ 10 h in primiparous women and ≥ 5 h in multiparous women. The pushing phase was defined prolonged if the duration was ≥ 60 min in primiparous women and ≥ 30 min in multiparous women. These cut offs were similar to the 75th quartiles of time in labor for the present study population; for primiparous women the 75th percentile was 13.0 h and for multiparous women the 75th percentile was 6.2 h (Table S2). Incorrect values of the labor time estimates were excluded. The time estimates were regarded incorrect, if they were < 11 min and ≥ 52 h for the total labor, < 10 min or ≥ 48 h for the active phase of first stage of labor and < 1 min and ≥ 4 h for the pushing phase, based the graphical distribution of time in labor excluding the outliers and based on clinical experience. Information on start of the pushing phase was not available for all women.

### Outcomes

The main outcome was maternal childbirth experience by VAS score. The VAS score was dichotomized into negative birth experience (VAS 1–3) and not being dissatisfied with childbirth (VAS 4–10). The definition of negative childbirth experience was based on the clinical recommendation at the participating study sites to offer extra psychologic support to the women scoring VAS 1–3^2^. The VAS score was further coded into three groups for descriptive analyses: negative birth experience (VAS 1–3), intermediate birth experience (VAS 4–7) and positive birth experience (VAS 8–10). The classification of positive birth experience as VAS 8–10 was based on the definition used in the national Swedish pregnancy register^29^.

### Statistics

Continuous data is presented as mean and one standard deviation (SD), or median and inter quartile range [IQR] if not normally distributed. Categorical data is presented as number and per cent. Differences in the categorical labor time variables between the VAS groups were analyzed with Chi-square test. The labor time variables were not normally distributed. Kruskal–Wallis test was used to compare median durations of the total active labor, the active phase in first stage of labor and the pushing phase among the three VAS groups. Multivariable logistic regression analyses were used to study the association between time in labor, in percentiles and categorical labor time variables, and binary outcomes; negative birth experience (VAS 1–3) and not being dissatisfied with childbirth (VAS 4–10), presented as crude and adjusted odds ratios (ORs and aORs). In the multivariable analyses, adjustments were made for maternal age, BMI, and fetal birthweight. The reference category for the analyses of the labor time estimates in primiparous and multiparous women was set as 10th–90th percentiles for duration of total active labor. In primiparous women the reference categories were set as < 12 h in total active labor, < 10 h in the active phase in first stage of labor, and < 60 min in the pushing phase. For multiparous women the reference categories were chosen as < 6 h in total active labor, < 5 h in the active phase in first stage of labor, and < 30 min in the pushing phase. The statistical analyses were performed using IBM SPSS version 26 (IMB inc, Armok, NY). A p-value < 0.05 was considered statistically significant.

### Sensitivity analyses

Sensitivity analyses were performed to examine the robustness of our findings.

First, we compared the prevalence of maternal characteristics and obstetric outcomes in the women that were excluded from the final study-population due to missing VAS score as well missing information on start of active labor, with the women included in the final study-population (Table S1).

Further we performed extended analyses on time in total active labor. Time in active labor was classified in < 25th percentile, 25th–75th percentile and > 75th percentile and sensitivity analyses examined whether the crude ORs for a negative childbirth experience differed compared to the main analyses (Table S2).

Sensitivity analyses of time in total active labor, classified in < 10th percentile, 10th–90th percentile and > 90th percentile and crude ORs for a negative childbirth experience in primiparous and multiparous women categorized according to type of onset of labor and mode of birth were done (Table S3,S4).

### Ethical approval

The Regional Ethical Review Board in Linköping, Sweden approved this study on October 26th, 2018 (Dnr 2018/337-31) and on January 1st, 2020 (Dnr 2019-04529). All methods were performed in accordance with the relevant guidelines and regulations.

## Results

**Figure 1:**
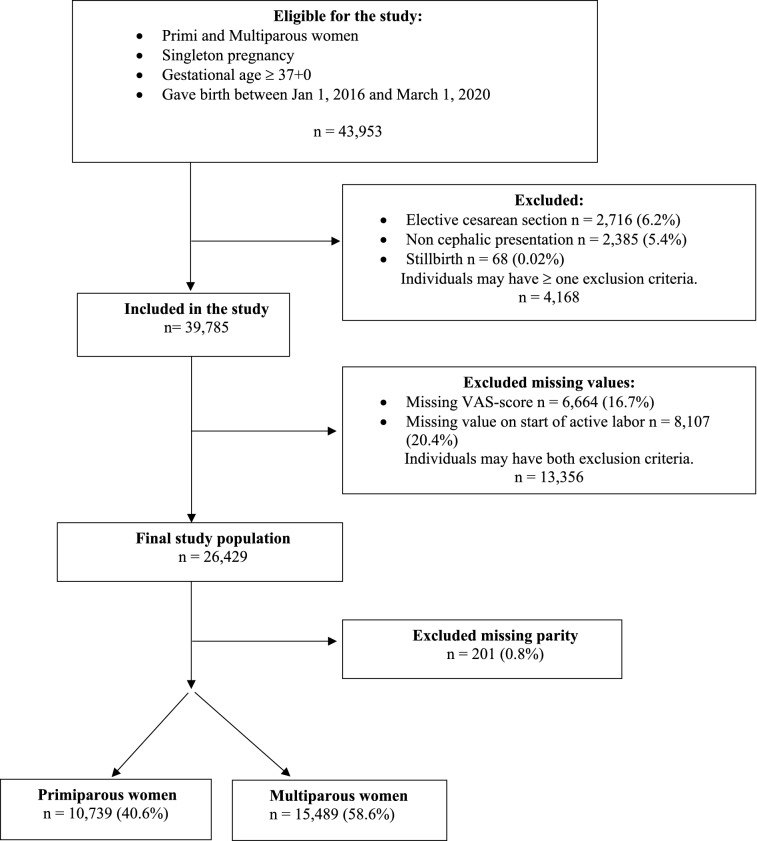
Flow chart of the study population.

A total number of 26,429 women, with a singleton term pregnancy, who had assessed their childbirth experience by VAS score and with known start of active labor constituted the final study population. Of all 43,953 eligible women, 9.5% (n = 4168) were excluded due to elective cesarean section, non-cephalic presentation and/or stillbirth. Of all 39,785 included women 33.6% (n = 13,356) were excluded due to missing information on VAS and/or start of active labor (Fig. 1).

In the final study population, 82.7% had a documented VAS score. Overall, 69.7% (*n* = 18,428) of the women had a positive birth experience (VAS 8–10), whereas 4.9% (*n* = 1298) reported a negative birth experience (VAS 1–3).

The maternal characteristics, obstetric interventions, and outcomes in the study-population, categorized according to a positive, intermediate, or negative childbirth experience, are presented in Table 1. The women who had a negative childbirth experience were statistically significantly older, primiparous, at gestational age ≥ 41 weeks, had induced labor, an infant with Apgar score < 7 at 5 min or with a birthweight of ≥ 4.5 kg, compared with the women in the total study population. Further, the frequencies of epidural anesthesia, oxytocin augmentation, operative vaginal birth or emergency CS, occurrence of obstetric anal sphincter injury and postpartum hemorrhage ≥ 1000 ml were statistically significantly higher among women with a negative childbirth experience (Table 1, p < 0.05 for all variables, not shown in Table 1).

**Table 1:** Maternal characteristics, obstetric interventions, and outcomes in women with categorized childbirth experiences (VAS).

	Total study population n = 26,429 n (%)	VAS 1–3 (negative childbirth experience)	VAS 4–7 (intermediate childbirth experience)	VAS 8–10 (positive childbirth experience)
**Maternal age (years)** **Mean [SD]**		30.6 [4.8]	30.2 [4.7]	30.0 [4.8]
n (%)	26,295 (99.5)			
< 25	3593 (13.6)	143 (11.1)	865 (12.9)	2585 (14.1)
25–29.9	10,130 (38.3)	491 (38.0)	2585 (38.7)	7054 (38.5)
30–34.9	8508 (32.2)	430 (33.3)	2161 (32.3)	5917 (32.3)
≥ 35	4064 (15.4)	227 (17.6)	1071 (16.0)	2766 (15.1)
**BMI (kg/m**^**2**^**)** **Mean [SD]**		25.6 [5.00]	25.3 [4.89]	25.2 [4.84]
n (%)	25,742 (97.4)			
< 18.5	605 (2.3)	20 (1.6)	142 (2.2)	443 (2.5)
18.5–24.9	13,919 (52.7)	644 (51.0)	3571 (54.5)	9704 (54.1)
25–29.9	7231 (27.4)	382 (30.3)	1803 (27.5)	5046 (28.1)
30–34.9	2811 (10.6)	146 (11.6)	729 (11.1)	1936 (10.8)
35–39.9	881 (3.3)	51 (4.0)	232 (3.5)	598 (3.3)
≥ 40	295 (1.1)	19 (1.5)	76 (1.2)	200 (1.1)
**Parity** **n (%)**	26,228 (99.2)			
Primipara	10,739 (40.6)	748 (58.3)	3216 (48.3)	677 (37.0)
Multipara	15,489 (58.6)	534 (41.7)	3442 (51.7)	11,513 (63.0)
**Gestational age (weeks)** **n (%)**				
37 + 0–39 + 6	10,416 (39.4)	448 (34.5)	2507 (37.4)	7461 (40.5)
40 + 0–40 + 6	8924 (33.8)	431 (33.2)	2307 (34.4)	6186 (33.6)
41 + 0–41 + 6	5393 (20.4)	308 (23.7)	1391 (20.8)	3694 (20.0)
≥ 42 + 0	1696 (6.4)	111 (8.6)	498 (7.4)	1087 (5.9)
**Onset of labor** **n (%)**				
Induction	3947 (14.9)	294 (22.7)	1038 (16.2)	2570 (13.9)
Spontaneous	22,482 (85.1)	1004 (77.3)	5620 (83.8)	15,858 (86.1)
Epidural anesthesia n (%)	10,846 (41.0)	836 (64.4)	3323 (49.6)	6687 (36.3)
Oxytocin augmentation n (%)	11,321 (42.9)	885 (68.2)	3452 (51.5)	6984 (37.9)
**Mode of birth** **n (%)**				
Non-instrumental vaginal birth	24,149 (91.4)	933 (71.9)	5782 (86.3)	17,434 (94.6)
Instrumental vaginal birth	1418 (5.4)	189 (14.5)	571 (8.5)	658 (3.6)
Emergency CS	862 (3.3)	176 (13.6)	350 (5.2)	336 (1.8)
**OASI **n (%)	616 (2.3)	78 (6.0)	223 (3.3)	315 (1.7)
**PPH ≥ 1000 ml** n (%)	1401 (5.3)	150 (11.7)	487 (7.3)	764 (4.2)
**Apgarscore < 7 at 5 min** n (%)	216 (0.8)	43 (3.3)	8 (1.2)	92 (0.5)
**Birthweight ≥ 4.5 kg** n (%)	879 (3.3)	64 (4.9)	266 (4.0)	549 (3.0)

Categorical data are presented as number and (%) and continuous data as mean and [SD].

*VAS* visual analogue scale, *BMI* body mass index, *CS* cesarean section, *OASI* obstetric anal sphincter injury, *PPH* postpartum hemorrhage.

The distribution of labor duration in primiparous and multiparous women according to a positive, intermediate, or negative childbirth experience, is presented by percentiles in Table 2. When analyzing the distribution of total labor duration in primiparous and multiparous women, according to the different labor duration percentiles, the same pattern emerges regardless of parity. The associations between the labor time categories defining prolonged labor and childbirth experience in primiparous and multiparous women are shown in Table 2. The proportion of primiparous and multiparous women with prolonged labor phases increased with decreasing VAS scores, indicating a negative childbirth experience (p for homogeneity < 0.001). Almost half of the primiparous women with a negative childbirth experience had a prolonged total active labor (48%) and in multiparous women with a negative childbirth experience 40% had a prolonged total active labor (Table 2).

**Table 2:** Duration of labor and childbirth experience in primiparous and multiparous women.

		Total study-population n	VAS 1–3 (negative childbirth experience) n (%)	VAS 4–7 (intermediate childbirth experience) n (%)	VAS 8–10 (positive childbirth experience n (%)	p-value
**Primiparous women**		10,730				
**Total active labor**						
Median (h)		9.17	11.70	9.95	8.48	< 0.001*
Percentiles	hours					
< 10	< 3.82	1056	32 (4.3)	272 (8.5)	752 (11.1)	
10–90		8600	555 (74.3)	2542 (79.1)	5503 (81.3)	
> 90	> 17.22	1074	160 (21.4)	398 (12.4)	516 (7.6)	
< 25	< 5.87	2695	111 (14.9)	708 (22.0)	1876 (27.7)	
25–75		5348	327 (43.8)	1554 (48.4)	3467 (51.2)	
> 75	> 13.02	2687	309 (41.4)	950 (29.6)	1428 (21.1)	
Time categories						
< 12 h			388 (51.9)	2066 (64.3)	4982 (73.6)	< 0.001
≥ 12 h			359 (48.1)	1146 (35.7)	1789 (26.4)	
**Active phase in first stage of labor**						
Median [IQR] (h)		8.08 [6.73]	10.48 [7.32]	8.83 [7.07]	7.67 [6.42]	< 0.001*
< 10 h			269 (47.0)	1622 (57.9)	4126 (66.3)	< 0.001
≥ 10 h			303 (53.0)	1177 (42.1)	2094 (33.7)	
**Pushing phase**						
Median [IQR] (min)		33.00 [29.00]	36.00 [34.00]	34.00 [30.00]	32.00 [29.00]	< 0.001*
< 60 min			440 (76.9)	2293 (81.9)	5260 (84.6)	< 0.001
≥ 60 min			132 (23.1)	507 (18.1)	955 (15.4)	
**Multiparous women**		15,477				
**Total active labor**						
Median (h)		3.83	4.98	4.20	3.72	< 0.001*
Percentiles	hours					
< 10	< 1.43	1449	34 (6.4)	278 (8.1)	1137 (9.9)	
10–90		12,486	393 (73.3)	2708 (78.7)	9385 (81.6)	
> 90	> 9.20	1542	106 (19.9)	454 (13.2)	982 (8.5)	
< 25	< 2.32	3820	95 (17.8)	757 (22.0)	2968 (25.8)	
25–75		7816	235 (44.1)	1654 (48.1)	5927 (51.5)	
> 75	> 6.18	3841	203 (38.1)	1029 (29.9)	2609 (22.7)	
Time ca﻿tegories						
< 6 h			320 (60.0)	2346 (68.2)	8716 (75.8)	< 0.001
≥ 6 h			213 (40.0)	1094 (31.8)	2788 (24.2)	
**Active phase in first stage of labor**						
Median [IQR] (h)		3.52 [3.70]	4.30 [4.68]	3.82[4.11]	3.47 [3.57]	< 0.001*
< 5 h			265 (58.1)	1961 (63.4)	7407 (69.3)	< 0.001
≥ 5 h			191 (41.9)	1134 (36.6)	3275 (30.7)	
**Pushing phase**						
Median [IQR] (min)		11.00 [13.00]	14.00 [19.00]	12.00 [15.00]	11.00 [13.00]	< 0.001*
< 30 min			362 (79.2)	2626 (84.9)	9572 (89.6)	< 0.001
≥ 30 min			95 (20.8)	468 (15.1)	1113 (10.4)	

*VAS* visual analogue scale, *h* hours, *min* minutes.

Categorical data are presented as number and (%) and continuous data as median [IQR].

P-values < 0.05 were considered statistically significant. *P-value for Kruskal–Wallis test.

Tables 3 and 4 demonstrate a significant association between duration of labor and risk of negative childbirth experience in primiparous and multiparous women, in all labor time categories that were analyzed. The analyses were adjusted for maternal age, BMI and fetal birthweight (Model 1 in Tables 3,4). For primiparous women with duration of total active labor above the 90th percentile the risk of a negative birth experience was more than doubled (aOR 2.39 (1.98–2.90), Model 1, Table 3). If, on the other hand, duration of total active labor was below the 10th percentile, primiparous women had a significantly reduced risk of a negative childbirth experience (aOR 0.46, 95% CI (0.32–0.69) (Table 3, Model 1).

**Table 3:** Time in labor and risk of negative childbirth experience (VAS 1–3) in primiparous women.

	Total study-population n (%) n = 10,739	Negative childbirth experience (VAS 1–3)			
		Number n (%)	Crude OR (95% CI)	Model 1* aOR (95% CI)	Model 2* aOR (95% CI)
**Total active labor**	10,730 (99.9)	747 (7.0)			
Percentiles					
< 10	1056	32 (4.3)	0.45 (0.32–0.65)	0.46 (0.32–0.69)	0.48 (0.33–0.70)
10–90	8600	555 (74.3)	Reference	Reference	Reference
> 90	1074	160 (21.4)	2.54 (2.10–3.07)	2.39 (1.98–2.90)	1.77 (1.44–2.17)
Time categories					
< 12 h	7436 (69.2)	388 (51.9)	Reference	Reference	Reference
≥ 12 h	3294 (30.7)	359 (48.1)	2.22 (1.91–2.58)	2.11 (1.81–2.46)	1.72 (1.46–2.02)
**Active phase in first stage of labor**	9591 (89.3)				
< 10 h	6017 (62.7)	269 (47.0)	Reference	Reference	Reference
≥ 10 h	3574 (37.3)	303 (53.0)	1.99 (1.67–2.35)	1.89 (1.59–2.24)	1.89 (1.59–2.24)
**Pushing phase**	9587 (89.3)				
< 60 min	7993 (83.4)	440 (76.9)	Reference	Reference	Reference
≥ 60 min	1594 (16.6)	132 (23.1)	1.55 (1.27–1.90)	1.49 (1.22–1.83)	1.26 (1.02–1.56)

*VAS* visual analogue scale, *h* hours, *min* minutes, *OR* odds ratio, *CI* confidence interval.

*Adjustments in.

Model 1: maternal age, BMI and fetal birthweight.

Model 2: maternal age, BMI, fetal birthweight, start of labor (spontaneous vs induction) and mode of delivery (vaginal birth vs cesarean section).

**Table 4:** Time in labor and risk of negative childbirth experience (VAS 1–3) in multiparous women.

	Total study-population n (%) n = 15,489	Negative childbirth experience (VAS 1–3)			
		Number n (%)	Crude OR (95% CI)	Model 1* aOR (95% CI)	Model 2* aOR (95%CI)
**Total active labor**	15,477 (99.9)	533 (3.4)			
Percentiles					
< 10	1449	34 (6.4)	0.74 (0.52–1.02)	0.74 (0.51–1.06)	0.73 (0–51-1.05)
10–90	12,486	393 (73.3)	Reference	Reference	Reference
> 90	1542	106 (19.9)	2.27 (1.82–2.83)	2.23 (1.78–2.79)	1.79 (1.40–2.28)
Time categories					
< 6 h	11,382 (73.5)	320 (60.0)	Reference	Reference	Reference
≥ 6 h	4095 (26.5)	213 (40.0)	1.90 (1.59–2.26)	1.91 (1.59–2.28)	1.68 (1.39–2.03)
**Active phase in first stage of labor**	14,233 (91.9)	456 (3.2)			
< 5 h	9633 (67.7)	265 (58.1)	Reference	Reference	Reference
≥ 5 h	4600 (32.3)	191 (41.9)	1.53 (1.27–1.85)	1.55 (1.28–1.88)	1.54 (1.27–1.88)
**Pushing phase**	14,236 (91.9)	457 (3.2)			
< 30 min	12,560 (88.2)	362 (79.2)	Reference	Reference	Reference
≥ 30 min	1676 (11.8)	95 (20.8)	2.03 (1.61–2.55)	2.05 (1.62–2.60)	1.89 (1.48–2.42)

*VAS* visual analogue scale, *h* hours, *min* minutes, *OR* odds ratio, *CI* confidence interval.

*Adjustments in.

Model 1: maternal age, BMI and fetal birthweight.

Model 2: maternal age, BMI, fetal birthweight, start of labor (spontaneous vs induction) and mode of delivery (vaginal birth vs cesarean section).

In primiparous women with a defined prolonged total labor (≥ 12 h) the risk of a negative childbirth experience was more than twice as high, compared with women with normal duration of labor; aOR 2.11, 95% CI (1.81–2.46). Primiparous women with prolonged active phase (≥ 10 h) had an increased risk for a negative childbirth experience; aOR1.89, 95% CI (1.59–2.24). The risk of negative childbirth experience was also increased (aOR 1.49, 95% CI (1.22–1.83)), if the pushing phase was ≥ 60 min (Table 3, Model 1).

For multiparous women with duration of total active labor above the 90th percentile, the risk of a negative childbirth experience was more than twice as high (aOR 2.23 (1.78–2.79), Model 1, Table 4). Further with a defined prolonged total active labor (≥ 6 h) and prolonged active phase (≥ 5 h) the risk of a negative childbirth experience was increased in multiparous women compared with women with normal duration of labor; aOR 1.91, 95% CI (1.59–2.28) and aOR1.55, 95%CI (1.28–1.88) respectively. A prolonged pushing phase in multiparous women doubled the risk of a negative birth experience aOR 2.05, 95%CI (1.62–2.60) (Table 4, Model 1).

When the multivariable logistic regression analyses on the association between time in labor and childbirth experience also included adjustments for onset of labor and mode of delivery, the aORs were still statistically significant and only changed marginally (Tables 3, 4, Model 2).

The sensitivity analyses comparing the prevalence of maternal characteristics and obstetric outcomes in women with available or missing VAS score as well as women with available or missing start of active labor demonstrated statistically significant differences. More women with gestational age ≥ 42 weeks, induction of labor and cesarean delivery had missing information on start of active labor compared to women with known start of active labor (Table S1). A larger proportion of women with missing VAS (7.9%) was also delivered by CS compared to women with known VAS (4.6%).

A dose–response relation between time in active labor and risk of a negative childbirth experience was seen, with statistically significant higher crude ORs for a negative childbirth experience when prolonged time in active labor was defined above the 90th percentile for both primiparous (OR 2.54 95% CI (2.10–3.07)) and multiparous women (OR 2.27 95% CI (1.81–2.83)) compared to the analyses when the 75th percentile of total labor duration was analyzed for primiparous (OR 2.00 95% CI (1.70–2.35) and multiparous women (OR 1.80 95% CI (1.49–2.81) (Table S2).

In Tables S3 and S4 the crude OR for negative birth experience for women, categorized according to type of start of labor and mode of delivery in percentiles of labor duration, are presented. Primiparous women with induced labor and labor duration above the 90th percentile, had higher crude OR for negative birth experience; OR 3.60 95% CI (2.42–5.35) compared to women with spontaneous onset of labor and labor duration above the 90th percentile; OR 2.34 95% CI (1.88–2.91) (Table S3). When comparing primiparous women with induction and spontaneous onset in labor time categories, the women with prolonged total labor (≥ 12 h) and induced labor had higher crude OR for a negative birth experience; OR 2.70 95% CI (1.88–3.88), than the women with spontaneous start and same categorization of prolonged labor; OR 1.87 95% CI (1.55–2.25) (OR not shown in Table S3). No difference was seen between the non-instrumental and instrumental vaginal delivery groups (Table S3). For multiparous women no significant differences were seen between women with induced labor and spontaneous start of labor or between women with instrumental and non-instrumental vaginal delivery (Table S4). For primi- and multiparous women delivered with CS, no statistically significant differences were seen (Tables S3, S4).

## Discussion

In this large multi-center population-based cohort study, we found a significant association between longer duration of active labor and low VAS score, indicating a negative childbirth experience in both primiparous and multiparous women and the most pronounced risk of a negative childbirth experience was seen for those with induced labor. All different labor phases that were studied were significantly related to the women’s childbirth satisfaction score. The longer duration of total labor, active phase and pushing phase, respectively, the lower both primiparous women and multiparous women rated their childbirth satisfaction score by VAS. The dose–response relation between time in active labor and risk of negative childbirth experience, with statistically significant higher crude ORs for a negative childbirth experience when prolonged labor was defined at or above the 90th percentile compared to the 75th percentile, strengthens the result that there is an association between long time in labor and an overall negative childbirth experience by VAS.

In line with previous research, the women in our cohort with negative childbirth experience were more likely to be primiparous, have induced labor, an infant with Apgar score < 7 at 5 min, oxytocin augmentation, operative vaginal birth or emergency CS, obstetric anal sphincter injury or postpartum hemorrhage^2,3,13^.

There are some studies that have investigated the relationship between prolonged labor and birth experience, although the definition of prolonged labor was not specified in all studies^30,31^. A Swedish study found that nulliparous women with negative birth experience reported longer labors measured in hours than women with positive birth experience^30^. An Iranian study stated that labor dystocia was a strong predictor of low birth satisfaction^31^. Our results are consistent with previous studies concluding that prolonged labor, defined as > 12 h, increased the risk of negative childbirth experience^14,19^. However, these studies were conducted at a single center, included smaller study populations, were restricted to primiparous women, and did not study the active phase of first stage of labor and the pushing phase separately^14,19^. In contrast to these studies, Fenaroli et al. found that the duration of first stage of labor did not affect the woman’s birthing experience among 111 Italian primiparous women who completed the Wijma Delivery Experience Questionnaire (W-DEQ), however a longer pushing phase contributed to a negative childbirth experience^23^. An association between prolonged second stage of labor and a negative childbirth experience, evaluated more than a decade after delivery was also found in primi- and multiparous American women who delivered by CS but not for those who delivered vaginally^6^. In another single center study including 70 primiparous women, Turkmen et al. showed that the childbirth satisfaction score by Childbirth Experience Questionnaire was not affected by the duration of neither the latent phase nor the active phase of labor^24^. An aspect when studying time in labor and women’s childbirth experiences is that women´s perceptions of start of active labor, the labor phases and time in labor might differ from the established medical definitions^32,33^. However, in a Swedish study, women with negative childbirth experiences both reported longer labors measured in hours and experienced longer labors and viewed the length as prolonged compared to women with a positive birth experience^34^.

A major strength of this study is the multicenter population-based design with prospectively recorded data in standardized medical records, which reduces the risk of selection bias and recall bias. There were some statistically significant differences between the women with missing or known start of active labor or VAS. We cannot exclude the possibility that the missing data may have had some influence on the adjusted estimates. To exclude a group of women that differ from the included women will influence the generalizability. There was a higher prevalence of women with gestational age ≥ 42 weeks and induction in the group with unknown start of active labor compared to women with known start of active labor. This might be explained by the fact that a gestational week ≥ 42 is an indication for induction of labor and some women with induction of labor never reach start of active labor before they are delivered by cesarean section due to failure to induce labor. This may also explain why more women with missing start of active labor (11.4%) were delivered by cesarean section than women with available start of active labor (3.6%). However, it is also possible that among the women that were excluded due to unknown start of active labor there were women with emergency CS due to slow progress of labor. Among the women in the final study population the percentage of women with labor duration above the 90th percentile was higher among women with CS compared with women with non-instrumental vaginal delivery. Hence, most likely, if these excluded women would answer similar to the women in the final study population, the estimates would be even more significant if more women with longer labor duration would have been included, following the hypothesis that women with prolonged labor are more likely to have negative birth experience. This strengthens the results.

To our knowledge, this study has the largest cohort of women with detailed information on maternal characteristics, known time in active labor and assessed childbirth experience, which gave sufficient power to evaluate the association between duration of the different labor phases and childbirth experience and in addition, enabled adjustments for possible confounding factors. If adjustments for all possible known confounders had been done, this might have resulted in different results. However, our purpose with this study was to evaluate the overall effect of time in labor and childbirth-experience focusing on the main groups primiparous and multiparous women, not necessarily to imply causality between the exposure and the outcome. Our approach could be looked upon as both a strength and a limitation.

In contrast to Kempe et al. who only analyzed the mean VAS score in relation to time in labor^14^, we categorized the VAS scores. To categorize the VAS scale into negative and positive childbirth experience and to present the distribution of exposure, time in labor, in percentiles gives a wider dimension of the childbirth experience compared with the mean value. Further, the high response rate of VAS (82.7%), increases the likelihood of representative study samples. The prevalence of negative childbirth experience of 4.9% is similar to the reported prevalence in previous studies^2,13^ which also strengthens the generalizability of the study results.

The evaluation of the overall childbirth experience by VAS score is an accessible, easy, and valid method that correlates with other birthing experience instruments such as the W-DEQ and the Childbirth Experience Questionnaire^24–26^. The VAS method is used nationwide in Sweden to assess women’s birth experiences a few days after childbirth and the VAS scores are registered in the national Swedish Pregnancy Register^2,3,14,35^. Another advantage of the VAS method is that it is part of the clinical routine and reaches the majority of all parturients and therefore is a good method to study a larger cohort.

On the other hand, the VAS method is a limitation due to its simplified and non-specific measure on childbirth experience overall that does not give a deeper understanding of the multifaceted childbirth experience. Some risk factors for a negative childbirth experience, such as personal experience of pain or support during labor^5,19,36^, was not documented in the women’s electronic medical records and can thus not be adjusted for in the analyses. This may decrease the validity of the present study. Further, there is no established definition of negative childbirth experience by VAS and another definition might have given different results. However, our definition of an overall negative childbirth experience by a low VAS score (1–3) was based on the current clinical guidelines at the participating study sites to offer extra psychosomatic support to women scoring below 4 and thereby including the women who were the most dissatisfied with their childbirth experience. Another limitation is that the childbirth experience was evaluated within the first 72 h after childbirth, since women’s rating of childbirth might be influenced by the initial positive feelings shortly after birth^37^. A certain amount of time estimates for the different phases of labor were missing, however there is no reason to suspect an association between missing time estimates and VAS that could have biased the results.

The definition of normal labor progression is currently under international debate. The WHO and ACOG has changed their definitions and allow a slower progress in the beginning of the active phase of labor^1,17^. The reason for this change has mainly been to reduce the incidence of emergency CS and avoid unnecessary emergency CS due to failure to progress. However, since long time in labor increases the risk of a negative birthing experience, as demonstrated in this study, it may also increase future demands on CS in the following pregnancy due to fear of childbirth^9,10^. Since a negative birth experience also may have every day and life-long consequences affecting the bonding with the newborn and the women’s mental health and result in avoidance of a future pregnancy as a result from fear of childbirth, care givers must pay attention to the women with long time in active labor^34,38,39^. The childbirth experience is multidimensional. Some contributing factors for a negative childbirth experience, like unexpected events such as instrumental delivery, emergency CS, and postpartum hemorrhage, are difficult to prevent, while other factors might be noticed and thus improve the care of women in labor. It is described that women who experienced more pain than expected, did not receive the support by the care givers that they needed, had insufficient continuous information and did not feel included in decisions made during labor were at risk of a negative childbirth experience^5,19,36^. Reducing the risk of these known factors might compensate for the effect of a longer time in labor. Hence, it might be of extra importance to make sure that women with longer time in labor have enough pain relief and to be aware of a possible need for extra support and guidance during labor. A cesarean delivery due to failure of labor progress might contribute to a negative childbirth experience^2,4,6,14,15^. It is therefore important not to terminate labor with CS due to failure of progress before the women have been offered continuous support and the expectations from the women have been ascertained*.* A Swedish study including 10 women with prolonged labor concluded that emotional support and encouragement by caregivers helped to accept the prolonged labor^40^. Clear information on labor interventions like induction procedure or instrumental delivery may also reduce the risk of negative birth experience^41^.

In conclusion, our data show that a longer time in active labor significantly increases the risk of an overall negative childbirth experience for both primiparous and multiparous women. The most pronounced risk of a negative childbirth experience was seen for those with induced labor. This risk also applies to the active phase of first stage of labor and the pushing phase separately. It is of great importance to identify and optimize the clinical care of women with long time in labor to reduce the risk of negative childbirth experience and associated adverse long-term effects.

## Supplementary Information

Supplementary Tables.

## Data Availability

The data that support the findings of this study are available on request from the corresponding author.

## Abbreviations

WHO: World Health Organization
CS: Cesarean section
ACOG: American College of Obstetricians and Gynecologists
VAS: Visual analog scale
BMI: Body mass index
OR: Odds ratio
aOR: Adjusted odds ratio
