# Supplementary material for: Maternal childbirth experience and time in labor: a population-based cohort study
Source: Sci Rep. 2022 Jul 13;12:11930. doi: 10.1038/s41598-022-14711-y (PMC9279318; doi:10.1038/s41598-022-14711-y)
Supplement: Supplementary file 1 — Supplementary Tables. [file 41598_2022_14711_MOESM1_ESM.docx]

**Supplementary Information file (Table S1-S4).**

**Title: Maternal childbirth experience and time in labor - a population-based cohort study**

Sara Carlhäll*^1^, MD, PhD; Marie Nelson^1^ CNM; Maria Svenvik^3^ MD; Daniel Axelsson^2^ MD, PhD; Marie Blomberg^1^, MD, PhD.

^1^ Department of Obstetrics and Gynecology in Linköping, Department of Biomedical and Clinical Sciences, Linköping University, Sweden

^2^ Department of Obstetrics and Gynecology, Ryhov County Hospital, Jönköping, Sweden and Department of Clinical and Experimental Medicine, Linköping University, Linköping, Sweden.

^3^ Department of Obstetrics and Gynecology, Region Kalmar County, Kalmar, Sweden, and Department of Biomedical and Clinical Sciences, Linköping University, Linköping, Sweden.

**Table S1. Sensitivity analyses on maternal characteristics and obstetric outcomes in women with available or missing VAS score and available or missing start of active labor.**

|  | **Available VAS**  **(n= 33,121)** | **Missing VAS**  **(n= 6,664)** | **Available start of active labor**  **(n= 31,678)** | **Missing start of active labor**  **(n=8,107)** |
| --- | --- | --- | --- | --- |
| **Maternal age (years)** | n (%) | n (%) | n (%) | n (%) |
| < 25 | 4,529 (13.9) | 1,106 (17.1) | 4,419 (14.2) | 1,216 (15.4) |
| 25-29.9 | 12,306 (37.8) | 2,224 (34.5) | 11,661 (37.5) | 2,869 (36.3) |
| 30-34.9 | 10,474 (32.2) | 1,954 (30.3) | 9,975 (32.1) | 2,453 (31.0) |
| ≥ 35 | 5,210 (16.0) | 1,165 (18.1) | 5,010 (16.1) | 1,365 (17.3) |
| **BMI** **(kg/m^2^)** |  |  |  |  |
| <18.5 | 648 (2.0) | 163 (2.6) | 662 (2.2) | 149 (1.9) |
| 18,5-24.9 | 16,878 (53.3) | 3,090 (49.9) | 16,198 (53.6) | 3,770 (49.2) |
| 25-29.9 | 9,090 (28.7) | 1,829 (29.5) | 8,596 (28.5) | 2,323 (30.3) |
| 30-34.9 | 3,524 (11.1) | 747 (12.1) | 3,297 (10.9) | 974 (12.7) |
| ≥ 35 | 1,531 (4.8) | 366 (5.9) | 1,458 (4.8) | 439 (5.7) |
| **Parity** |  |  |  |  |
| Primipara | 13,491(41.7) | 2,323 (36.5) | 12,504 (40.5) | 3,310 (42.1) |
| Multipara | 18,890 (58.3) | 4,050(63.5) | 18,8385 (59.5) | 4,555 (57.9) |
| **Gestational age (weeks)** |  |  |  |  |
| 37+0-39+6 | 13,012 (39.6) | 2,692 (40.7) | 12,450 (39.6) | 3,254 (40.5) |
| 40+0-40+6 | 10,923 (33.2) | 2,107 (31.8) | 10,566 (33.6) | 2,464 (30.6) |
| 41+0-41+6 | 6,603 (20.1) | 1,328 (20.1) | 6,418 (20.4) | 1,513 (18.8) |
| ≥ 42+0 | 2,357 (7.2) | 494 (7.5) | 2,038 (6.5) | 813 (10.1) |
| **Onset of Labor** |  |  |  |  |
| Induction | 5,633 (17.0) | 1,204 (18.1) | 4,790 (15.1) | 2,047 (25.2) |
| Spontaneous | 27,488 (83.0) | 5,460 (81.9) | 26,888 (84.9) | 6,060 (74.8) |
| **Epidural anesthesia** | 13,765 (41.6) | 2,556 (38.4) | 12,897 (40.7) | 3.424 (42.2) |
| **Mode of birth** |  |  |  |  |
| Non-instrumental vaginal birth | 29,766 (89.9) | 5,701 (85.5) | 28,782 (90.9) | 6,685 (82.5) |
| Instrumental vaginal birth | 1,828 (5.6) | 438 (6.6) | 1,767 (5.6) | 499 (6.1) |
| Emergency CS | 1,527 (4.6) | 525 (7.9) | 1,129 (3.6) | 923 (11.4) |
| **OASI** | 788 (2.4) | 196 (2.9) | 773 (2.4) | 211 (2.6) |
| **PPH ≥** **1000 ml** | 1904 (5.7) | 434 (6.5) | 1,745 (5.5) | 593 (7.3) |
| **Apgar score < 7 at 5 min** | 304 (0.9) | 191(2.9) | 331 (1.0) | 164 (2.0) |
| **Birthweight ≥** **4,5 kg** | 1,165 (3.5) | 246 (3.7) | 1,062 (3.4) | 349 (4.3) |

VAS = visual analogue scale, BMI = body mass index, CS = cesarean section, OASI = obstetric anal sphincter injury, PPH = Postpartum hemorrhage. Categorical data are presented as number and (%).

**Table S2. Duration of total active labor and risk of negative childbirth experience (VAS 1-3) in primiparous and multiparous women.**

|  | **Primiparous women (n = 10,730)** | | | | **Multiparous women (n =15,477)** | | | |
| --- | --- | --- | --- | --- | --- | --- | --- | --- |
| **Total active labor (percentiles)** | **Time (h)** | **Total**  **n (%)** | **VAS 1-3**  **n (%)** | **Crude OR (95% CI)** | **Time (h)** | **Total**  **n (%)** | **VAS 1-3**  **n (%)** | **Crude OR (95% CI)** |
| < 25 | <5.87 | 2,695(25.1) | 111(14.9) | 0.66(0.53-0.82) | < 2.32 | 3,820 (24.7) | 95(17.8) | 0.82(0.65-1.05) |
| 25-75 |  | 5,348(48.9) | 327(43.8) | reference |  | 7,816 (50.5) | 235(44.1) | reference |
| >75 | >13.02 | 2,687(25.0) | 309(41.4) | 2.00(1.70-2.35) | >6.18 | 3,841 (24.8) | 203(38.1) | 1.80(1.49–2.81) |

VAS = visual analogue scale; h = hours; OR = odds ratio; CI = confidence interval.

**Table S3. Sensitivity analyses of time in total active labor and risk of negative childbirth experience (VAS 1-3) in primiparous women according to type of onset of labor and mode of birth.**

|  | **Onset of labor** | | | | | | **Mode of birth** | | | | | | | | | | | |
| --- | --- | --- | --- | --- | --- | --- | --- | --- | --- | --- | --- | --- | --- | --- | --- | --- | --- | --- |
|  | **Spontaneous**  **n = 9,129** | | | **Induction**  **n = 1,601** | | | **Non-Instrumental vaginal delivery**  **n = 9,068** | | | **Instrumental vaginal delivery**  **n = 1,076** | | | **Vaginal delivery (non-instrumental and instrumental)**  **n = 10,144** | | | **Cesarean section**  **n = 574** | | |
| **Total active labor**  **(per-centiles)** | **Total**  **n (%)** | **VAS**  **1-3**  **n (%)** | **Crude OR (95% CI)** | **Total**  **n (%)** | **VAS 1-3**  **n (%)** | **Crude OR (95% CI)** | **Total**  **n (%)** | **VAS 1-3**  **n (%)** | **Crude OR (95% CI)** | **Total**  **n (%)** | **VAS 1-3**  **n (%)** | **Crude OR (95% CI)** | **Total**  **n (%)** | **VAS 1-3**  **n (%)** | **Crude OR (95% CI)** | **Total**  **n (%)** | **VAS 1-3 n (%)**  **132** | **Crude OR (95% CI)** |
| <10 | 817 (8.9) | 21 (3.7) | 0.43 (0.28-0.67) | 239 (14.9) | 11 (6.0) | 0.41 (0.22-0.76) | 987 (10.9) | 24 (5.2) | 0.47 (0.31-0.71) | 4 (3.9) | 1 (0.7) | NA | 1,029 (10.1) | 25 (4.1) | 0.41 (0.27-0.61) | 27 (4.7) | 7 (5.3) | 1.31 (0.53-3.22) |
| 10-90 | 7,385 (80.9) | 426 (75.7) | ref | 1,215 (75.9) | 129 (70.1) | ref | 7,423 (81.9) | 376 (81.9) | ref | 811 (75.4) | 99 (66.0) | ref | 8,234 (81.2) | 475 (78.0) | ref | 356 (62.0) | 75 (56.8) | ref |
| >90 | 927 (10.1) | 116 (20.6) | 2.34 (1.88-2.91) | 147 (9.2) | 44 (23.9) | 3.60  (2.42-5.35) | 658 (7.3) | 59 (12.9) | 1.85 (1.39-2.46) | 223 (20.7) | 50 (33.3) | 2.02 (1.39-2.94) | 881 (8.7) | 109 (17.9) | 2.31  (1.85-2.88) | 191 (33.2) | 50 (37.9) | 1.33 (0.88-2.00) |

VAS= visual analogue scale; OR= odds ratio; CI= confidence interval.

**Table S4. Sensitivity analyses of time in total active labor and risk of negative childbirth experience (VAS 1-3) in multiparous women according to type of onset of labor and mode of birth.**

|  | **Onset of labor** | | | | | | **Mode of birth** | | | | | | | | | | | |
| --- | --- | --- | --- | --- | --- | --- | --- | --- | --- | --- | --- | --- | --- | --- | --- | --- | --- | --- |
|  | **Spontaneous**  **n = 13,162** | | | **Induction**  **n = 2,315** | | | **Non-instrumental vaginal delivery**  **n = 14,890** | | | **Instrumental vaginal delivery**  **n = 308** | | | **Vaginal delivery (non-instrumental and instrumental)**  **n = 15,198** | | | **Cesarean Section**  **n = 278** | | |
| **Total active labor**  **(per-centiles)** | **Total**  **n (%)** | **VAS 1-3**  **n (%)** | **Crude OR (95% CI)** | **Total**  **n (%)** | **VAS 1-3**  **n (%)** | **Crude OR (95% CI)** | **Total**  **n (%)** | **Vas 1-3**  **n (%)** | **Crude OR (95% CI)** | **Total**  **n (%)** | **Vas 1-3 n (%)** | **Crude OR (95% CI)** | **Total**  **n (%)** | **VAS 1-3**  **n (%)**  **609** | **Crude OR (95% CI)** | **Total**  **n (%)** | **VAS**  **1-3**  **n (%)** | **Crude OR (95% CI)** |
| <10 | 1,048 (8.0) | 20 (4.7) | 0.65 (0.41-1.02) | 401 (17.3) | 14 (13.2) | 0.76 (0.42-1.35) | 1,434  (9.6) | 33 (7.1) | 0.77 (0.54-1.11) | 9 (2.9) | 0 (0) | NA | 1,443 (9.5) | 33 (6.7) | 0.75 (0.52-1.08) | 6 (2.2) | 1 (2.4) | NA |
| 10-90 | 10,712 (81.4) | 312 (73.1) | ref | 1,774 (76.6) | 81 (76.4) | ref | 12,155 (81.6) | 359 (77.4) | ref | 196 (63.6) | 14  (50.0) | ref | 12,351 (81.3) | 373 (75.8) | ref | 135 (48.6) | 20 (48.8) | ref |
| >90 | 1,402 (10.7) | 95 (22.2) | 2.42 (1.91-3.07) | 140 (6.0) | 11 (10.4) | 1.78 (0.93-3.43) | 1,301 (8.7) | 72 (15.5) | 1.93 (1.49-2.50) | 103 (33.4) | 14  (50.0) | 2.05 (0.94-4.47) | 1,404 (9.2) | 86 (17.5) | 2.10 (1.65-2.67) | 137 (49.3) | 20 (48.8) | 0.98 (0.50-1.92) |

VAS= visual analogue scale; OR= odds ratio; CI= confidence interval.
